# Impact of Renal Replacement Therapy on Mortality and Renal Outcomes in Critically Ill Patients with Acute Kidney Injury: A Population-Based Cohort Study in Korea between 2008 and 2015

**DOI:** 10.3390/jcm11092392

**Published:** 2022-04-24

**Authors:** Subin Hwang, Danbee Kang, Hyejeong Park, Youngha Kim, Eliseo Guallar, Junseok Jeon, Jung-Eun Lee, Wooseong Huh, Gee-Young Suh, Juhee Cho, Hye-Ryoun Jang

**Affiliations:** 1 Department of Internal Medicine, Seoul Paik Hospital, Inje University School of Medicine, Seoul 04551, Korea; subin.151719@gmail.com; 2 Center for Clinical Epidemiology, Samsung Medical Center, Sungkyunkwan University School of Medicine, Seoul 06531, Korea; dbee.kang@skku.edu (D.K.); hj.park219@sbri.co.kr (H.P.); youngha1223@gmail.com (Y.K.); eguallar@jhu.edu (E.G.); 3 Department of Clinical Research Design and Evaluation, Samsung Advanced Institute for Health Science and Technology, Sungkyunkwan University School of Medicine, Seoul 06531, Korea; 4 Department of Epidemiology and Medicine and Welch Center for Prevention, Epidemiology and Clinical Research, Johns Hopkins Medical Institutions, Baltimore, MD 21287, USA; 5 Division of Nephrology, Department of Medicine, Samsung Medical Center, Sungkyunkwan University School of Medicine, Seoul 06531, Korea; uncleimdr@gmail.com (J.J.); jungeun34.lee@samsung.com (J.-E.L.); wooseong.huh@samsung.com (W.H.); 6 Department of Critical Care Medicine, Samsung Medical Center, Sungkyunkwan University School of Medicine, Seoul 06351, Korea; smccritcare@gmail.com

**Keywords:** acute kidney injury, chronic kidney disease, continuous renal replacement therapy, end-stage of kidney disease, intermittent hemodialysis, mortality, renal replacement therapy

## Abstract

The outcomes depending on the type of renal replacement therapy (RRT) or pre-existing kidney disease in critically ill patients with acute kidney injury (AKI) have not been fully elucidated. All adult intensive care unit patients with AKI in Korea from 2008 to 2015 were screened. A total of 124,182 patients, including 21,165 patients with pre-existing kidney disease, were divided into three groups: control (no RRT), dialysis, and continuous RRT (CRRT). In-hospital mortality and progression to end-stage kidney disease (ESKD) were analyzed according to the presence of pre-existing kidney disease. The CRRT group had a higher risk of in-hospital mortality. Among the patients with pre-existing kidney disease, the dialysis group had a lower risk of in-hospital mortality compared to other groups. The risk of ESKD was higher in the dialysis and CRRT groups compared to the control group. In the CRRT group, the risk of ESKD was even higher in patients without pre-existing kidney disease. Although both dialysis and CRRT groups showed a higher incidence of ESKD, in-hospital mortality was lower in the dialysis group, especially in patients with pre-existing kidney disease. Our study supports that RRT and pre-existing kidney disease may be important prognostic factors for overall and renal outcomes in patients with AKI.

## 1. Introduction

Acute kidney injury (AKI) is a common complication of critically ill patients suffering from various diseases such as heart failure or septic shock and is associated with high mortality and progression to chronic kidney disease (CKD) [1,2]. Renal replacement therapy (RRT) is frequently required in patients with severe AKI [3]. Given the evident advances in critical care medicine, the application of RRT has significantly increased [4]. Although a number of studies have reported renal prognosis and mortality according to the modality of RRT, current clinical data do not support the superiority of specific RRT modalities [5]. The RRT modality is usually determined according to patients’ conditions and institutional availability of dialysis or continuous RRT (CRRT) equipment.

Moreover, although AKI is usually regarded as a consequence of serious conditions or a component of multiorgan failure in critically ill patients, the most important factor affecting renal outcomes may be the presence of an underlying kidney disease, which increases susceptibility to AKI or impairs renal recovery [6]. Several studies have reported that renal function after AKI affects long-term outcomes. An et al. [7] showed that renal functional assessment at 3 months after CRRT initiation can help predict progression to end-stage kidney disease (ESKD) and long-term survival. Further, a retrospective cohort study reported an estimated glomerular filtration rate of <30 mL/min per 1.73 m^2^ at hospital discharge after CRRT as a strong risk factor for poor long-term survival and a poor renal prognosis [8]. However, only a few recent studies have analyzed renal and patient outcomes after AKI depending on the modality of RRT and pre-existing kidney diseases in a representative large cohort of critically ill patients.

In this study, we analyzed a large database from a nationwide cohort including virtually all intensive care unit (ICU) admissions in Korea to investigate the impact of application and modality of RRT and pre-existing kidney diseases on in-hospital mortality, progression to ESKD, and length of stay (LOS) in hospitals and ICUs.

## 2. Materials and Methods

### 2.1. Data Source

We retrospectively analyzed the cohort of the Korean ICU National Data (KIND) study [9] based on the Health Insurance Review and Assessment (HIRA) database of the Korean Ministry of Health. This database includes all ICU admissions in Korea. The HIRA database contains health insurance claims data generated in the process of reimbursing healthcare services under the National Health Insurance (NHI) system in Korea. The HIRA database contains information on almost 50 million patients as of 2014 in Korea, covering 98% of the total population through the universal health coverage system [10]. The study was reviewed by the Institutional Review Board (IRB) of Samsung Medical Center (IRB protocol 2015-11-17). The informed consent was waived because only previously collected deidentified administrative data were used.

### 2.2. Study Population

The study population included all adult patients older than 18 years from the KIND study (*n* = 131,988), who were diagnosed with AKI during the first ICU admission from 1 January 2008 to 31 May 2015. These patients had no history of AKI, dialysis, CRRT treatment, or ICU admission within a year prior to hospitalization.

**Figure 1:**
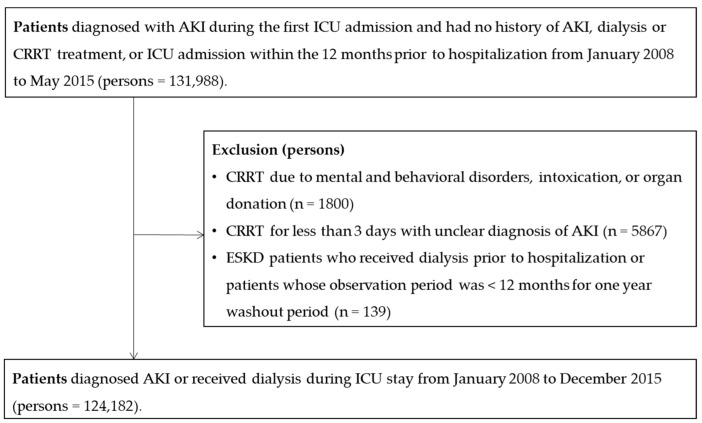
Flowchart of study participants (*n* = 124,182).

Patients with the following characteristics were excluded: (1) those who received CRRT due to mental and behavioral disorders, intoxication, or organ donation (*n* = 1800); (2) those who received CRRT for less than 3 days, which could not be confirmed as AKI because of the possibility of applying CRRT for other diseases such as drug intoxication (*n* = 5867); and (3) ESKD patients who received dialysis prior to hospitalization, or patients who had been observed for less than 12 months during a one-year washout period (*n* = 139). Finally, a total of 124,182 patients (73,512 men and 50,670 women) were included (Figure 1).

### 2.3. Study Variables

ICU admission was defined by examining the claim codes that all hospitals in Korea are required to use when they submit cost claims of ICU admissions to the HIRA service (codes AJ100-AJ590900). These codes are based on the Korean Classification of Diseases 6th edition, a modified version of the International Classification of Diseases 10th revision (ICD-10), adapted for use in the Korean health system [11]. All ICU stays during the same hospitalization were considered as a single ICU admission. Similarly, hospital stays separated by <2 days were considered the same hospital admission.

AKI was defined as the presence of codes that identified AKI (ICD-10 codes N17), RRT including CRRT (Korean NHI procedure codes O7031-O7035, O7051-O7055), or dialysis. Dialysis was defined as intermittent hemodialysis (O7020-O7021, O2011-O2012, O2081-O2083, or O9991) or peritoneal dialysis (O7061-O7062, O7071-O7075, E6581, E6582, or E6593) using Korean NHI procedure codes.

Initiation timing and the modality of RRT were decided considering patients’ overall conditions and RRT equipment status of each center. Indications for RRT were symptomatic uremia, severe electrolyte imbalance including hyperkalemia, severe metabolic acidosis, and volume overload according to guidelines [12,13]. CRRT was preferentially initiated in these patients with hemodynamic instability, multiorgan failure, or risk of increased intracerebral pressure.

We collected claim codes regarding information on comorbidities, management procedures during ICU admission, prescriptions, and demographic characteristics. Comorbidities, including pre-existing kidney disease within the year prior to hospitalization, were summarized using the Charlson index [14,15]. Kidney diseases were additionally defined using codes for chronic or unspecified nephritic syndromes (ICD-10 codes N030, N031, N038, N039, N050, N051, N058, or N059), which are not included in the Charlson Comorbidity Index.

The management procedures included mechanical ventilation (MV; Korean NHI procedure codes M5857, M5858, or M5860) and extracorporeal membrane oxygenation (ECMO, Korean NHI procedure codes O1901-O1904, material codes CAPIOX EBS CIRCUIT G5401008, QUADROX PLS G5501050, or CAPIOX EBS PMP CIRCUIT G5501008). Administration of inotropic or vasopressor drugs such as dobutamine, dopamine, epinephrine, and norepinephrine for more than 2 days was also identified using Korean drug and anatomical therapeutic chemical codes (148201BIJ, 38900BIJ, 148701BIJ, 148702BIJ, 429500BIJ, 152601BIJ, or 203101BIJ) [16].

We obtained information regarding hospital characteristics from the HIRA Medical Care Institution Database, which included the type of institution, location, number of hospital beds, facilities, and physicians. The type of hospital was classified according to the capacity, based on the number of hospital beds and the number of specialties as defined by the Korean Health Law [17]. In general, hospitals are defined as healthcare institutions with more than 30 inpatient beds. General hospitals are hospitals with more than 100 beds and more than seven specialty departments, including internal medicine, surgery, pediatrics, obstetrics and gynecology, anesthesiology, pathology, and laboratory medicine. Tertiary hospitals are general hospitals with more than 20 specialty departments that serve as teaching hospitals to medical students and nurses.

Total cost was the amount of money reimbursed by Korean NHI to hospitals, including ICU stay and for patients’ medical services endorsed by HIRA, and then was converted into US dollars using the exchange rate of 1 December 2015 (1158 won/dollar).

### 2.4. Definition of Outcomes

The primary outcome was in-hospital mortality, defined as the death code of the billing statement. We also compared hospital and ICU LOS (days) and the incidence of ESKD after discharge among survivors (*n* = 77,185). To define post-discharge outcomes, we linked the personal identification number of each study participant to the inpatient claims data in the admission result database. The progression to ESKD as the long-term outcome of AKI was evaluated between hospitalization and 1 year after discharge. ESKD was defined in patients who received dialysis for >3 months (codes O7020, O9991, O7075) with registration for a copayment decreasing policy for ESKD patients (codes V001 and V003) or patients who underwent kidney transplantation (codes R3280).

### 2.5. Statistical Analysis

All patients were divided into three groups according to the RRT modality: control (no RRT), dialysis, and CRRT groups. Patients who received both dialysis and CRRT were categorized into the CRRT group. Continuous variables are presented as mean and standard deviation (SD) or median and interquartile range (IQR) and compared using one-way analysis of variance (ANOVA). Categorical variables are presented as numbers and proportions and compared using the χ^2^ test.

Because patients’ outcomes could be clustered by a hospital [18], we used the hospital as a random intercept in mixed-effects logistic regression models to estimate odds ratios (ORs) with 95% confidence intervals (CIs).

Patients who died during hospitalization were excluded from the analyses of progression to ESKD, as well as from the hospital and ICU LOS analysis to avoid the inclusion of censored patients due to death. A total of 77,185 patients were included in the final analysis. For hospital and ICU LOS in survivors, we used multiple linear regression models to compare the three groups. Since hospital LOS and ICU LOS were markedly right-skewed, log_e_-transformed outcomes and the estimated ratio with 95% CI comparing the three groups were used.

The long-term outcome of this study was the incidence of ESKD between hospitalization and 1 year after discharge. Person-time was calculated from the date of hospital admission to ESKD or the last follow-up date. Survival curves were generated using the Kaplan–Meier product-limit method and compared using log-rank tests. We used Cox proportional hazards regression models to estimate the hazard ratio (HR) with 95% CI for ESKD. We examined the proportional hazards assumption using plots of the log (−log) survival function and Schoenfeld residuals.

Age, sex, type of hospital, history of comorbidities (myocardial infarction, congestive heart failure, peripheral vascular disease, cerebrovascular disease, rheumatologic disease, liver disease, diabetes mellitus, kidney disease, cancer, and acquired immune deficiency syndrome/human immunodeficiency virus), and the use of MV, vasopressor drugs, and ECMO were adjusted in the final model. In addition, we conducted a subgroup analysis to evaluate the association of RRT with each outcome, depending on pre-existing kidney diseases.

All analyses were two-sided, and *p*-values < 0.05 were considered statistically significant. Statistical analyses were performed using SAS version 9.2 (SAS Institute, Inc., Cary, NC, USA) and R software (version 3.3.2; Free Software Foundation, Inc., Boston, MA, USA).

## 3. Results

**Figure 2:**
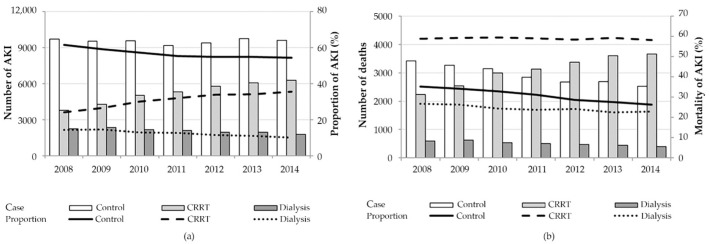
Yearly trends of acute kidney injury and in-hospital mortality according to renal replacement therapy modality: (**a**) Bars and lines represent the absolute numbers and the proportion of critically ill patients with AKI among all intensive care unit patients, respectively. The proportion of patients who received CRRT increased steadily from 24.1% in 2008 to 36.7% in 2014.; (**b**) Bars and lines represent the absolute number of deaths and the mortality of critically ill patients with AKI, respectively. In-hospital mortality rate of patients who received CRRT increased by 1% for 7 years (58.9% in 2008 to 59.7% in 2014).

A total of 124,182 ICU patients who were diagnosed with AKI were analyzed: 56.5%, 12.2%, and 31.3% in the control, dialysis, and CRRT groups, respectively (Table 1). The average proportion of AKI patients who received CRRT was 31.1% during the study period, which increased steadily from 24.1% in 2008 to 36.7% in 2014. During the same period, the in-hospital mortality rate of patients who received CRRT increased by 1% (58.9–59.7%) (Figure 2).

The mean patient age (SD) was 67.1 ± 15.2 years, and 59.2% of the patients were men. The dialysis and CRRT groups included younger patients (control vs. dialysis and CRRT: 69.4% vs. 64.5% and 64.0%, *p* < 0.001), were more likely to include males (57.4% vs. 60.1% and 62.0%, *p* < 0.001), had a higher proportion of pre-existing kidney diseases (11.0% vs. 39.3% and 19.2%, *p* < 0.001), and showed more frequent admission in tertiary hospitals (28.8% vs. 40.6% and 57.5%, *p* < 0.001) than the control group. Among the three groups, MV (80.3%), ECMO (5.1%), and vasopressor drugs (49.5%) were most frequently used in the CRRT group.

The in-hospital mortality rate was significantly higher in the CRRT group than in the control and dialysis groups (control vs. dialysis vs. CRRT; 30.8% vs. 24.4% vs. 59.0%; *p* < 0.001) (Table 2). Even after adjustment for several confounders, the CRRT group had a significantly higher in-hospital mortality than the control and dialysis groups (fully adjusted or compared with the control group 2.04 (95% CI, 1.98–2.11, *p* < 0.001)). When the association between RRT and in-hospital mortality was evaluated depending on the pre-existing kidney disease, the CRRT group showed a significantly higher risk of in-hospital mortality in both subgroups (fully adjusted or compared with the control group; 2.10 (95% CI, 2.03–2.17) in the subgroup without pre-existing kidney disease vs. 1.70 (95% CI, 1.56–1.84) in the subgroup with pre-existing kidney disease). In the dialysis group, the in-hospital mortality of the subgroup without pre-existing kidney disease was comparable with that of the control group (fully adjusted or compared with the control group 0.98 (95% CI, 0.93–1.03)). However, the dialysis subgroup with pre-existing kidney disease showed lower in-hospital mortality (fully adjusted OR compared with the control group 0.67 (95% CI, 0.61–0.74)).

The median hospital LOS among survivors (*n* = 77,185) was 17 days (IQR, 9–31), 25 days (IQR, 14–43), and 29 days (IQR, 16–53) in the control, dialysis, and CRRT groups, respectively (Table 3). The dialysis group was more likely to have a longer hospital and ICU LOS than the control group. ICU LOS in the CRRT group was 43% longer than that in the control group. The association between RRT and LOS was consistent regardless of pre-existing kidney diseases.

**Figure 3:**
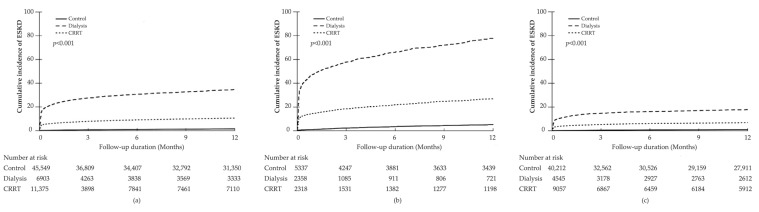
Cumulative incidence of end-stage kidney disease (ESKD) according to the modality of renal replacement therapy: All patients (**a**); patients with pre-existing kidney disease (**b**); and patients without pre-existing kidney disease (**c**); the risk of ESKD was the highest in the dialysis group with pre-existing kidney disease.

In all patients who were discharged, the median follow-up of AKI patients was 365 days (IQR 128–365 days). During the 47,141.1 person-years of follow-up period, 3433 (4.45%) patients progressed to ESKD (incidence rate 72.8/1000 person-years). The incidence rate of ESKD was higher in the dialysis and CRRT groups than in the control group (control vs. dialysis vs. CRRT groups; 16.2 vs. 463.8 vs. 126.5 per 1000 person-years). In the Kaplan–Meier survival analyses, the cumulative incidence of ESKD was significantly higher in the dialysis group than in the control and CRRT groups (log-rank test, *p* < 0.001) (Figure 3).

Compared with the control group, the age- and gender-adjusted HRs for ESKD were 25.48 (95% CI, 21.30–30.48) and 7.74 (95% CI, 6.59–9.10) in the dialysis and CRRT groups, respectively. This association remained significant after further adjustment for multiple confounders (fully adjusted HR compared with the control group: 17.67 (95% CI, 15.06–20.72) in the dialysis group and 7.28 (95% CI, 6.29–8.41) in the CRRT group) (Table 4). In patients with pre-existing kidney disease, the risk of ESKD in the dialysis and CRRT groups was higher than that in the control group (fully adjusted HR compared with the control group; dialysis vs. CRRT groups, 15.15 (95% CI, 12.88–17.83) vs. 5.83 (95% CI, 4.89–6.96); *p* < 0.001). In patients without pre-existing kidney disease, the risk of ESKD was even higher in both dialysis and CRRT groups than in the control group (fully adjusted HR; dialysis vs. CRRT groups, 20.06 (95% CI, 16.06–25.05) vs. 8.86 (95% CI, 7.30–10.76); *p* < 0.001).

## 4. Discussion

This study investigated the overall outcomes of AKI depending on the type of RRT and pre-existing kidney disease in critically ill adult patients using a nationwide population-based cohort in Korea during a 7-year period. The CRRT group had a significantly higher risk of in-hospital mortality, whereas the dialysis group showed lower in-hospital mortality than the control group. Specifically, among the patients without pre-existing kidney disease, in-hospital mortality was higher in the CRRT group, and the incidence of ESKD was also higher in both dialysis and CRRT groups than in the control group. These results suggest that severe AKI that develops as a consequence of multiorgan failure may exert a critically adverse impact on patient survival and renal outcomes. Further, the lower in-hospital mortality in the dialysis group may indicate the importance of appropriate RRT in the treatment of AKI in critically ill patients.

Previous prospective randomized studies comparing the effects of dialysis and CRRT showed no difference in mortality between the two treatment modalities [19,20,21]. One meta-analysis of 30 randomized controlled trials and eight prospective cohort studies found no difference in all-cause mortality between patients treated with intermittent hemodialysis and those treated with CRRT [22]. In a recent meta-analysis of 21 studies, the modality of RRT was not associated with in-hospital and ICU mortality [23]. According to a retrospective population-based matched cohort study comparing outcomes according to CRRT and hemodialysis (propensity-matched patients with no difference in mechanical ventilation, number of days between hospitalization and initiation of RRT, and baseline characteristics), there was no difference in mortality between the two groups [24]. In contrast to these reports, our results showed that the CRRT group had significantly higher mortality than both the control and dialysis groups. In clinical practice, CRRT has been preferentially used in AKI patients with unstable vital signs or poor overall medical conditions; thus, these patients are expected to show a higher mortality rate. Further, in an 8-year observational cohort study by De Corte et al., non-survivors had worse profiles of disease severity, and CRRT as the initial RRT modality was associated with long-term mortality [25]. In our study, although it was not possible to obtain severity scores directly from our database, ECMO, mechanical ventilation, and vasopressor were used more frequently in the CRRT group than in the dialysis or the control groups, suggesting that patients with higher severity scores were included in the CRRT group. Unlike other previous studies with no difference in mortality between the dialysis group and the CRRT group, the CRRT group in our study had a higher mortality. We believe that our result was more representative of real-world practice reflecting the overall severity of patients.

Few studies have investigated the impact of pre-existing kidney disease and RRT modality on AKI outcomes among critically ill patients. In our study, the dialysis group with pre-existing kidney disease showed lower in-hospital mortality than the control group. A prospective cohort study of AKI in critically ill patients reported that patients with pre-existing kidney disease had a lower mortality rate but were more likely to be dialysis-dependent at hospital discharge [26]. CKD is known to increase susceptibility to AKI, and AKI on CKD is associated with a worse renal outcome [27]. Patients with pre-existing kidney disease may take a relatively shorter time to reach the indications for RRT during the course of AKI, or early RRT can be considered based on their medical history of kidney disease. Our hypothesis is that multiorgan failure might be more frequent in AKI patients without pre-existing kidney disease, and RRT might be performed relatively earlier in patients with pre-existing kidney disease, resulting in reduced in-hospital mortality in our study population. Moreover, the mortality at day 60 was significantly higher in patients who received late RRT (61.8%) than in those who received early RRT (48.5%) in the Artificial Kidney Initiation in Kidney Injury (AKIKI) trial [28], which supports our hypothesis. The Early versus Late Initiation of Renal Replacement Therapy in Critically Ill Patients with AKI (ELAIN) trial also demonstrated that early initiation of RRT significantly reduced 90-day and 1-year all-cause mortality compared to delayed initiation of RRT [29,30].

Furthermore, intrarenal alterations at a molecular, cellular, and tissue level related to pre-existing kidney diseases seem to affect not only the severity of AKI but also the degree of the renal repair process, including fibrosis [6]. Previous studies reported that AKI contributes to the deterioration of renal function in CKD patients [31,32]. In a retrospective cohort study including patients older than 67 years, those with AKI on CKD showed a significantly higher risk of ESKD than those with AKI or CKD alone [26]. Further, in a cohort study of 9425 patients with postoperative AKI, patients with pre-existing kidney disease had higher risks of long-term mortality and dialysis dependency than those without pre-existing kidney disease [32]. Additionally, in several studies, the CRRT group showed better renal recovery and lower rate of dialysis dependency than the dialysis group [24,33,34,35,36]. Similarly, our results showed that the cumulative incidence of ESKD was higher in the dialysis group than in the CRRT group, especially in the subgroup with pre-existing kidney disease. The incidence rate of ESKD was significantly higher in patients with pre-existing kidney disease, regardless of the RRT modality received. However, the dialysis group showed lower in-hospital mortality than the control group. These results support the clinical importance of timely RRT to improve the survival of critically ill patients with AKI, although maintenance dialysis reduces the quality of life and increases the burden of medical costs [37]. Our results also suggest the necessity of further study focusing on overall outcomes of AKI in critically ill patients stratified by the presence of pre-existing kidney disease.

This study has several limitations. First, detailed clinical data, such as etiology of AKI, serum creatinine, and urine volume could not be analyzed because of the inherent limitations of the national registry database used in our study. However, the main purpose of this study was to compare hard outcomes such as in-hospital mortality and the risk of ESKD development depending on the application of RRT or RRT modalities. Although a contemporary consensus-based definition of AKI could not be used, we believe that our large database was sufficient to investigate the primary aim of our study. Second, the overall severity scores of critically ill patients, such as sequential organ failure assessment (SOFA) and acute physiology and chronic health evaluation (APACHE) scores, were not measured. However, considering that MV, ECMO, and vasopressor drugs were used more frequently in the CRRT group than in the dialysis group, the CRRT group seemed to include patients with hemodynamic instability and worse medical conditions. Unlike previous studies showing similar mortality in both CRRT and dialysis groups, our study showed higher mortality in the CRRT group after adjusting for confounding variables. We believe that our study reveals a difference in mortality among critically ill patients with AKI according to the modality of RRT in the real world. Third, the definition of ‘kidney disease’ was based on diagnostic codes. The NHIS department regularly audits claims in the Korean health insurance system, and the government provides special insurance coverage by evaluating the procedures and treatment under disease codes. Therefore, our HIRA database is considered reliable and has been widely used for research purposes [10]. 

Despite these limitations, our study has clinically important strengths including the study’s design and statistical methods based on the analysis of a large population-based cohort. This enables a meaningful report of mortality and progression to ESKD considering both available RRT modalities and the presence of pre-existing kidney disease. Additionally, medical treatments associated with relevant critically-ill patient conditions such as MV, ECMO, and vasopressor drugs were comprehensively analyzed.

## 5. Conclusions

Among critically ill patients with AKI in Korea, patients with pre-existing kidney disease in the dialysis group had relatively lower in-hospital mortality and a greater risk of progression to ESKD than those with pre-existing kidney disease in both the control and CRRT groups. In-hospital mortality was the highest in the CRRT group without pre-existing kidney disease, while the risk of ESKD was higher in the subgroup without pre-existing kidney disease. Our findings may support the critical impact of severe AKI requiring RRT on both mortality and long-term renal outcome in critically ill patients, including those without pre-existing kidney disease. Further prospective studies considering pre-existing kidney disease are required to elucidate the clinical impact of RRT modality on overall and renal outcomes in clinically ill patients with AKI.

## Figures and Tables

**Table 1:** Patient characteristics.

	Control	Dialysis	CRRT	*p*-Value
**Number of patients**	70,096	15,174	38,912	
**Gender**				<0.001
Male	40,259 (57.4)	9120 (60.1)	24,133 (62.0)	
Female	29,837 (42.6)	6054 (39.9)	14,779 (38.0)	
**Age, years**	69.4 (14.9)	64.5 (15.1)	64.0 (15.1)	<0.001
**Pre-existing kidney disease**	7720 (11.0)	5964 (39.3)	7481 (19.2)	<0.001
**Comorbidity**				
Myocardial infarction	4131 (5.9)	897 (5.9)	2444 (6.3)	0.03
Congestive heart failure	10,827 (15.4)	3006 (19.8)	5990 (15.4)	<0.001
Peripheral vascular disease	12,776 (18.2)	3108 (20.5)	7001 (18.0)	<0.001
Cerebrovascular disease	18,740 (26.7)	3598 (23.7)	8134 (20.9)	<0.001
Rheumatologic disease	3657 (5.2)	805 (5.3)	2516 (6.5)	<0.001
Liver disease	21,710 (31.0)	5135 (33.8)	15,127 (38.9)	<0.001
Diabetes	28,606 (40.8)	8355 (55.1)	17,908 (46.0)	<0.001
Cancer	10,527 (15.0)	2453 (16.2)	9306 (23.9)	<0.001
AIDS/HIV	51 (0.1)	8 (0.1)	36 (0.1)	0.279
**Type of hospital**				<0.001
Tertiary	20,157 (28.8)	6162 (40.6)	22,390 (57.5)	
General	46,978 (67.0)	8857 (58.4)	16,297 (41.9)	
Nursing care hospital	96 (0.1)	17 (0.1)	7 (0.0)	
Other	2865 (4.1)	138 (0.9)	218 (0.6)	
**Management procedures**				
Mechanical ventilation	26,662 (38.0)	5564 (36.7)	31,248 (80.3)	<0.001
ECMO	271 (0.4)	25 (0.2)	2002 (5.1)	<0.001
Vasopressor drugs	15,868 (22.6)	3206 (21.1)	19,278 (49.5)	<0.001
Total cost, USD 10 *	490 (229–971)	807 (433–1459)	1450 (720–2805)	<0.001

Values are numbers and proportions, except for age, Charlson index (mean and standard deviation), and total cost (median and interquartile range). CRRT, continuous renal replacement therapy; ECMO, extracorporeal membrane oxygenation. Dialysis was defined as intermittent hemodialysis and peritoneal dialysis. * USD 1 = KRW 1158 (exchange rate as of 1 December 2015).

**Table 2:** Risk of hospital mortality according to RRT modalities and pre-existing kidney disease.

	Control	Dialysis	CRRT	*p*-Value
**Number of patients**	70,096	15,174	38,912	
**Gender**				<0.001
Male	40,259 (57.4)	9120 (60.1)	24,133 (62.0)	
Female	29,837 (42.6)	6054 (39.9)	14,779 (38.0)	
**Age, years**	69.4 (14.9)	64.5 (15.1)	64.0 (15.1)	<0.001
**Pre-existing kidney disease**	7720 (11.0)	5964 (39.3)	7481 (19.2)	<0.001
**Comorbidity**				
Myocardial infarction	4131 (5.9)	897 (5.9)	2444 (6.3)	0.03
Congestive heart failure	10,827 (15.4)	3006 (19.8)	5990 (15.4)	<0.001
Peripheral vascular disease	12,776 (18.2)	3108 (20.5)	7001 (18.0)	<0.001
Cerebrovascular disease	18,740 (26.7)	3598 (23.7)	8134 (20.9)	<0.001
Rheumatologic disease	3657 (5.2)	805 (5.3)	2516 (6.5)	<0.001
Liver disease	21,710 (31.0)	5135 (33.8)	15,127 (38.9)	<0.001
Diabetes	28,606 (40.8)	8355 (55.1)	17,908 (46.0)	<0.001
Cancer	10,527 (15.0)	2453 (16.2)	9306 (23.9)	<0.001
AIDS/HIV	51 (0.1)	8 (0.1)	36 (0.1)	0.279
**Type of hospital**				<0.001
Tertiary	20,157 (28.8)	6162 (40.6)	22,390 (57.5)	
General	46,978 (67.0)	8857 (58.4)	16,297 (41.9)	
Nursing care hospital	96 (0.1)	17 (0.1)	7 (0.0)	
Other	2865 (4.1)	138 (0.9)	218 (0.6)	
**Management procedures**				
Mechanical ventilation	26,662 (38.0)	5564 (36.7)	31,248 (80.3)	<0.001
ECMO	271 (0.4)	25 (0.2)	2002 (5.1)	<0.001
Vasopressor drugs	15,868 (22.6)	3206 (21.1)	19,278 (49.5)	<0.001
Total cost, USD 10 *	490 (229–971)	807 (433–1459)	1450 (720–2805)	<0.001

Values are numbers and proportions, except for age, Charlson index (mean and standard deviation), and total cost (median and interquartile range). CRRT, continuous renal replacement therapy; ECMO, extracorporeal membrane oxygenation. Dialysis was defined as intermittent hemodialysis and peritoneal dialysis. * USD 1= KRW 1158 (exchange rate as of 1 December 2015).

**Table 3:** Hospital and ICU length of stay according to RRT modalities and pre-existing kidney disease.

	Length of Stay, Days	Crude Model		Adjusted Model ^a^		
	Median (IQR)	Ratio ^b^ (95% CI)	*p*-Value	Ratio ^b^ (95% CI)	*p*-Value	
**Hospital length of stay**						
**Overall**						
Control	17 (9–31)	Reference		Reference		
Dialysis	25 (14–43)	1.40 (1.37–1.43)	<0.001	1.46 (1.43–1.49)	<0.001	
CRRT	29 (16–53)	1.45 (1.42–1.48)	<0.001	1.22 (1.2–1.25)	<0.001	
**Pre-existing kidney disease**						
**With pre-existing kidney disease**						
Control	16 (9–30)	Reference		Reference		
Dialysis	24 (14–41)	1.42 (1.37–1.47)	<0.001	1.48 (1.43–1.53)	<0.001	
CRRT	28 (16–48)	1.57 (1.51–1.64)	<0.001	1.35 (1.3–1.41)	<0.001	
**Without pre-existing kidney disease**						
Control	17 (9–32)	Reference		Reference		
Dialysis	25 (15–46)	1.44 (1.40–1.48)	<0.001	1.46 (1.42–1.50)	<0.001	
CRRT	30 (16–55)	1.43 (1.40–1.46)	<0.001	1.19 (1.16–1.22)	<0.001	
**ICU length of stay**						
**Overall**						
Control	6 (2–14)	Reference		Reference		
Dialysis	6 (3–15)	0.95 (0.93–0.97)	<0.001	1.10 (1.08–1.13)	<0.001	
CRRT	18 (8–34)	2.06 (2.02–2.11)	<0.001	1.43 (1.40–1.46)	<0.001	
**Pre-existing kidney disease**						
**With pre-existing kidney disease**						
Control	5 (2–12)	Reference		Reference		
Dialysis	5 (2–10)	0.88 (0.85–0.92)	<0.001	0.98 (0.94–1.01)	0.239	
CRRT	14 (6–28)	2.18 (2.08–2.28)	<0.001	1.52 (1.45–1.58)	<0.001	
**Without pre-existing kidney disease**						
Control	6 (2–14)	Reference		Reference		
Dialysis	8 (4–20)	1.13 (1.10–1.17)	<0.001	1.20 (1.17–1.23)	<0.001	
CRRT	18 (8–37)	2.10 (2.05–2.15)	<0.001	1.40 (1.37–1.43)	<0.001	

ICU, intensive care unit; RRT, renal replacement therapy; CRRT, continuous renal replacement therapy; IQR, interquartile range; CI, confidence interval. ^a^ Adjusted for differences in type of hospital, history of comorbidity (myocardial infarction, congestive heart failure, peripheral vascular disease, cerebrovascular disease, rheumatologic disease, liver disease, diabetes, kidney disease (adjusted only overall group), cancer, AIDS/HIV), mechanical ventilator, vasopressor drugs, and extracorporeal membrane oxygenation. ^b^ Ratios and 95% CI were estimated from linear regression with log transformed variable as the outcome.

**Table 4:** Risk of end-stage kidney disease (ESKD) according to RRT modalities and pre-existing kidney disease.

	Person Years	No. of Cases	Incidence Rate (per 1000 Person-Years)	Model 1 HR (95% CI)	Model 2 HR (95% CI)
**Overall**					
Control	35,140.5	570	16.2	Reference	Reference
Dialysis	3986.5	1849	463.8	25.48 (21.30–30.48)	17.67 (15.06–20.72)
CRRT	8014.1	1014	126.5	7.74 (6.59–9.10)	7.28 (6.29–8.41)
*p*-value				<0.001	<0.001
**With pre-existing kidney disease**					
Control	3984.4	222	55.7	Reference	Reference
Dialysis	974	1168	1199.2	15.76 (13.37–18.58)	15.15 (12.88–17.83)
CRRT	1426.5	480	336.5	5.37 (4.52–6.38)	5.83 (4.89–6.96)
*p*-value				<0.001	<0.001
**Without pre-existing kidney disease**					
Control	31,156.1	348	11.2	Reference	Reference
Dialysis	3012.5	681	226.1	19.22 (15.39–24.01)	20.06 (16.06–25.05)
CRRT	6587.6	534	81.1	7.30 (6.03–8.85)	8.86 (7.30–10.76)
*p*-value				<0.001	<0.001

RRT, renal replacement therapy; CRRT, continuous renal replacement therapy. Model 1 was adjusted for age and gender. Model 2 was further adjusted for type of hospital, history of comorbidity (myocardial infarction, congestive heart failure, peripheral vascular disease, cerebrovascular disease, rheumatologic disease, liver disease, diabetes, kidney disease (adjusted only overall group), cancer, AIDS/HIV), mechanical ventilator, vasopressor drugs, and extracorporeal membrane oxygenation.

## Data Availability

The datasets used and/or analyzed in this study are available from the corresponding author upon reasonable request.
